# Linderane Suppresses Hepatic Gluconeogenesis by Inhibiting the cAMP/PKA/CREB Pathway Through Indirect Activation of PDE 3 via ERK/STAT3

**DOI:** 10.3389/fphar.2018.00476

**Published:** 2018-05-15

**Authors:** Wei Xie, Yangliang Ye, Ying Feng, Tifei Xu, Suling Huang, Jianhua Shen, Ying Leng

**Affiliations:** ^1^State Key Laboratory of Drug Research, Shanghai Institute of Materia Medica, Chinese Academy of Sciences, Shanghai, China; ^2^University of Chinese Academy of Sciences, Beijing, China

**Keywords:** cAMP, gluconeogenesis, linderane, phosphodiesterase, type 2 diabetes

## Abstract

The role of phosphodiesterase 3 (PDE3), a cyclic AMP (cAMP)-degrading enzyme, in modulating gluconeogenesis remains unknown. Here, linderane, a natural compound, was found to inhibit gluconeogenesis by activating hepatic PDE3 in rat primary hepatocytes. The underlying molecular mechanism and its effects on whole-body glucose and lipid metabolism were investigated. The effect of linderane on gluconeogenesis, cAMP content, phosphorylation of cAMP-response element-binding protein (CREB) and PDE activity were examined in cultured primary hepatocytes and C57BL/6J mice. The precise mechanism by which linderane activates PDE3 and inhibits the cAMP pathway was explored using pharmacological inhibitors. The amelioration of metabolic disorders was observed in *ob/ob* mice. Linderane inhibited gluconeogenesis, reduced phosphoenolpyruvate carboxykinase (*Pck1*) and glucose-6-phosphatase (*G6pc*) gene expression, and decreased intracellular cAMP concentration and CREB phosphorylation in rat primary hepatocytes under both basal and forskolin-stimulated conditions. In rat primary hepatocytes, it also increased total PDE and PDE3 activity but not PDE4 activity. The suppressive effect of linderane on the cAMP pathway and gluconeogenesis was abolished by the non-specific PDE inhibitor 3-isobutyl-1-methylxanthine (IBMX) and the specific PDE3 inhibitor cilostazol. Linderane indirectly activated PDE3 through extracellular regulated protein kinase 1/2 (ERK1/2) and signal transducer and activator of transcription 3 (STAT3) activation. Linderane improved glucose and lipid metabolism after chronic oral administration in *ob/ob* mice. Our findings revealed linderane as an indirect PDE3 activator that suppresses gluconeogenesis through cAMP pathway inhibition and has beneficial effects on metabolic syndromes in *ob/ob* mice. This investigation highlighted the potential for PDE3 activation in the treatment of type 2 diabetes.

## Introduction

Persistent hyperglycemia, the most notable characteristic of T2DM, contributes to a group of chronic complications and comorbidities (Saltiel, 2001). Abnormal elevation of hepatic gluconeogenesis is a major contributing factor in the onset of hyperglycemia in diabetic subjects (Gastaldelli et al., 2000). Metformin, the first-line drug for diabetes treatment, was reported to improve glucose homeostasis mainly through inhibition of gluconeogenesis, although its mechanism of action has not been thoroughly defined (Jones, 2016). The development of active molecules to inhibit gluconeogenesis would be an appealing strategy for glycemic control in patients with diabetes.

The cAMP signaling pathway plays a critical role in regulating energy homeostasis at multiple levels, including mediating hepatic gluconeogenesis (Yang and Yang, 2016). Physiologically, hormones such as glucagon and glucocorticoid elevate the production of cellular cAMP (Corvera et al., 1984; Imai et al., 1993). Then, cAMP binds to the regulatory subunit of PKA and triggers its migration to the nucleus. The catalytic subunit of PKA phosphorylates CREB at Ser 133, and the activated CREB dephosphorylates and recruits cAMP-modulated transcriptional co-activators (CRTCs). This process upregulates the expression of rate-limited gluconeogenic genes including *Pck1* (also known as *Pepck*) and *G6pc* (also known as *G6Pase*), then enhances hepatic gluconeogenesis and finally increases the whole-body blood glucose levels (Herzig et al., 2001; Samuel and Shulman, 2016). Metformin was reported to decrease the production of cAMP by upregulating the cellular AMP level and suppressing AC activity, which partly explained its ability to inhibit hepatic gluconeogenesis (Miller et al., 2013). In addition, two alkaloids, lycoricidine and lycoricidinol, were discovered to inhibit endogenous hepatic glucose production by suppressing cAMP-regulated CREB phosphorylation (Yun et al., 2016). This evidence indicated that interfering with the cAMP/PKA/CREB signaling pathway would be an attractive strategy to correct dysregulated hepatic glucose production in T2DM (Unger and Cherrington, 2012).

Degradation of cAMP is important in maintaining the intracellular cAMP concentration. Cyclic nucleotide PDEs are primarily responsible for catalyzing cAMP hydrolysis, and there are 11 families (PDE1–11) that share a range of catalytic activities in multiple tissues. In the liver, two types of PDEs, PDE3 and PDE4, are mainly responsible for degrading cAMP (Francis et al., 2011). However, the potential regulatory effect of hepatic PDEs on glucose metabolism, especially gluconeogenesis, is unclear. PDE4B was reported to be activated by AMPK in the liver (Johanns et al., 2016), but its role in hepatic glucose metabolism has remained largely unknown (Zhang et al., 2009). PDE3B-null mice showed elevated cAMP accumulation and enhanced gluconeogenesis in the liver, but the contribution of the specific depletion of hepatic PDE3B activity has not been identified (Choi et al., 2006). Overall, these studies indicated that PDEs might be involved in the regulation of hepatic gluconeogenesis by catalyzing cAMP hydrolysis. However, to elucidate the precise function of PDEs in the regulation of gluconeogenesis, pharmacological PDE activators are needed.

In the present study, we identified the natural product linderane as a PDE3 indirect activator. Linderane is a major bioactive component in Linderae, a widely used Chinese herb (Wu et al., 2010) that has been demonstrated to possess multiple biological effects, including superoxide anion radical-scavenging and antioxidative activity and protective activity against gastritis, gastric ulcers and backache (Duke and Ayensu, 1985; Noda and Mori, 2007; Wang et al., 2015). A previous study showed that linderane could protect human hepatoma HepG2 cells from H_2_O_2_-induced oxidative damage (Wu et al., 2010). However, its beneficial effect on hepatic energy control has never been reported. Here, we first discovered that linderane could activate PDE3 in hepatocytes and suppress hepatic gluconeogenesis by inhibiting the cAMP/PKA/CREB pathway. Moreover, the underlying molecular mechanism by which linderane activates PDE3 was investigated.

## Materials and Methods

### Animals

Male Sprague-Dawley (SD) rats and male C57BL/6J mice were purchased from SLAC Laboratory Animals (Shanghai, China), and fed with a normal diet (consisting of 20.5 kcal% protein, 4.62 kcal% fat, and 52.5 kcal% carbohydrates, Cat.M02-F, SLAC Laboratory Animals). B6.V-*Lep^ob^/Lep^ob^* (*ob/ob*) mice (Jackson Laboratory, Bar Harbor, ME, United States) were bred at the Shanghai Institute of Materia Medica (SIMM), Chinese Academy of Sciences (CAS), and fed with a high fat diet (consisting of 18.8 kcal% protein, 16.2 kcal% fat, and 45.2 kcal% carbohydrates, Cat.M04-F, SLAC Laboratory Animals). All animals were maintained in a 12 h light/12 h darkness cycle with free access to chow and water. Animal experiments were conducted in accordance with guides by Institutional Animal Care and Utilization Committee (IACUC), Shanghai Institute of Materia Medica (SIMM), Chinese Academy of Sciences (CAS). The protocol was approved by IACUC, SIMM, CAS.

### Culture of Rat Primary Hepatocytes

Primary hepatocytes were isolated from a male SD rat by Seglen’s two-step technique using a previously described protocol (Collins et al., 2006) with modifications. Briefly, an SD rat was fasted for 24 h to deplete glycogen in the liver. After anesthesia (sodium pentobarbital, 50 mg kg^-1^), the liver was perfused with collagenase digestion medium, and the hepatocytes were collected after 100 μm mesh filtration and Percoll (GE Healthcare, Uppsala, Sweden) centrifugation. Isolated hepatocytes were plated in collagen-coated 6- or 48-well plates (1 or 0.125 × 10^6^ cells per well) in minimum essential medium (MEM) supplemented with fetal bovine serum (10%, vol/vol, Gibco), insulin (100 nM, Sigma-Aldrich, St. Louis, MO, United States), and dexamethasone (10 nM, Sigma-Aldrich) and cultured at 37°C and 5% CO_2_ in a humidified incubator.

### Gluconeogenesis

Rat primary hepatocytes were plated in collagen-coated plates for 4 h before experiments. Gluconeogenic precursors were added to evaluate the effect of test compounds on hepatic gluconeogenesis. Linderane was bought from Pufei De Biotech (Chengdu, China). The purity is 98%. It was solved in DMSO and stored at -20°C. The final concentration of DMSO in culture media was 0.1%. For the experiments conducted without pharmacological inhibitors, rat primary hepatocytes were incubated in serum-free, glucose-free Dulbecco’s modified Eagle’s medium (DMEM) supplemented with metformin (500 μM, Sigma-Aldrich) or different doses of linderane for 1.5 h, followed by 4 h of incubation in the presence or absence of gluconeogenic substrates (2 mM sodium pyruvate, plus 20 mM sodium lactate, Sigma-Aldrich) with or without the stimulation of forskolin (20 μM, Sigma-Aldrich). For the experiments conducted with pharmacological inhibitors, hepatocytes were pretreated with a specific inhibitor for 0.5 h and then co-treated with metformin (500 μM) or different doses of linderane for 1.5 h. Subsequently, hepatocytes were incubated in the presence of both inhibitors and metformin or linderane with or without gluconeogenic substrates for 4 h. Medium was collected to determine glucose production, which was measured by a glucose assay kit (Rongsheng Biotech, Shanghai, China) and normalized to cellular protein concentration.

### Real-Time PCR

Total RNA was extracted with TRIzol reagent (Life Technologies, Carlsbad, CA, United States) according to the manufacturer’s instructions. cDNA was generated by a PrimerScript^TM^ RT reagent kit with gDNA eraser (perfect real time) (TaKaRa Biotechnology, Dalian, China). Target gene mRNA was quantified using SYBR Premix Ex Taq (TaKaRa). All oligonucleotide primers were purchased from Invitrogen, and the sequences are shown in **Table 1**. Gene expression was normalized against *Rn18s* (for samples from hepatocytes) or *Actb* (for samples from liver tissues) control RNA.

**Table 1:** mRNA primer sequences used for real-time PCR.

	Genes	Forward sequences (5′ to 3′)	Reverse sequences (5′ to 3′)
Rat	*Pck1*	TGACATTGCCTGGATGAAGT	GTCTTAATGGCGTTCGGATT
	*G6pc*	GACTCCCAGGACTGGTTTGT	GATGCCCACAGTCTCTTGAA
	*Rn18s*	CACGGGTGACGGGGAATCAG	CGGGTCGGGAGTGGGTAATTTG
Mouse	*Pck1*	CATATGCTGATCCTGGGCATAAC	CAAACTTCATCCAGGCAATGTC
	*G6Pc*	ACACCGACTACTACAGCAACAG	CCTCGAAAGATAGCAAGAGTAG
	*Actb*	TGACAGGATGCAGAAGGAGA	GCTGGAAGGTGGACAGTGAG


### cAMP Assay

The cAMP level in rat primary hepatocytes or liver was measured using an ELISA kit purchased from Enzo Life Sciences (Farmingdale, NY, United States).

### Western Blot Analysis

Protein preparation and western blot analysis were performed as described previously (Hu et al., 2007). Briefly, samples were separated by SDS-PAGE and transferred to PVDF membranes (Bio-Rad, Hercules, CA, United States). The blots were blocked with non-fat dry milk (7.5%) in Tris-buffered saline/Tween 20 (TBST) for 2 h and then incubated with primary antibodies at 4°C overnight. Antibodies against p-CREB Ser133 (#9198), CREB (#9197), ERK1/2 (#4695), p-ERK1/2 Thr202/Tyr204 (#4370), p-STAT3 Tyr705 (#9145), STAT3 (#4904), and GAPDH (#5174) were purchased from Cell Signaling Technology (Danvers, MA, United States) and diluted at 1:1000 for use. The horseradish peroxidase-conjugated secondary antibodies (Dingguo, Beijing, China) were used at 1:20,000 dilutions for GAPDH and 1:5000 otherwise. Immunoreactive proteins were detected by ECL plus Western blotting detection reagent (GE Healthcare, Buckinghamshire, United Kingdom) and quantified by densitometry (Bio-Rad).

### PDE Activity Measurement

Phosphodiesterase activity was detected by a PDE activity assay kit according to the manufacturer’s instructions (Abcam, Cambridge, United Kingdom) based on the modified Malachite Green method. The cAMP hydrolyzing activity measured in this method represents the activity of both PDEs and nucleotide cleavage enzymes like 5′-nucleotidase (Feng et al., 2011). Purified PDE enzymes were applied to detect the direct effect, and cell lysates from hepatocytes or livers were applied to detect the indirect effect on PDE activity. For detecting the total PDE activity, IBMX was applied as a pan-PDE inhibitor. The samples were incubated with cAMP substrates, 5′-Nase, and with or without IBMX for 30 min at 30°C. End the process with green assay buffer and read the microplate at 620 nm. The difference between the cAMP hydrolyzing activity with IBMX and without IBMX was the total PDE activity. In the liver, PDE3 and PDE4 account for the majority of PDE activity (Hermsdorf and Dettmer, 1998). For detecting PDE3 activity, the PDE3 specific inhibitor cilostazol was used. The samples were incubated with cAMP substrates, 5′-Nase, and with or without cilostazol. The cAMP hydrolyzing activity in the absence of cilostazol minus the activity in the presence of cilostazol was the PDE3 activity. For detecting PDE4 activity, the PDE4 specific inhibitor roflumilast was used. The samples were incubated with cAMP substrates, 5′-Nase, and with or without roflumilast. The cAMP hydrolyzing activity in the absence of roflumilast minus the activity in the presence of roflumilast was the PDE4 activity.

### Linderane Treatment in C57BL/6J Mice

Linderane (50 mg kg^-1^) or vehicle (0.25% sodium carboxyl methyl cellulose, CMC-Na) was administered orally to overnight fasted C57BL/6J mice (male, 8–9 weeks). After anesthesia (sodium pentobarbital, 50 mg kg^-1^), livers were dissected 2 h later for PDE activity, cAMP measurement, and western blot analysis.

### Linderane Treatment in *ob/ob* Mice

Linderane (50 mg kg^-1^) or vehicle (0.25% CMC-Na) was administered orally twice daily to male *ob/ob* mice (6- to 7-weeks-old) for 20 days. The effect of linderane on metabolic abnormalities was investigated. Briefly, male *ob/ob* mice were assigned to two groups based on blood glucose level and body weight (*n* = 8). Linderane (50 mg kg^-1^) or vehicle (0.25%, CMC-Na) was administered twice daily by oral gavage for 20 days. The random-fed and fast blood glucose levels were measured at day 8, 12, 16, and 20 by an ACCU-CHEK Advantage II glucose monitor. Body weight was detected regularly throughout the whole treatment. On the last day, the mice were anesthetized by an intraperitoneal injection of sodium pentobarbital (50 mg kg^-1^) after 6 h of fasting, and blood samples were collected. The liver was dissected, weighed, and stored at -80°C. Serum triacylglycerol (TG) was determined by commercial kits purchased from Zhejiang Dongou Diagnostics Co., Ltd. (Wenzhou, China). HbA_1c_ was measured using kits from Roche Diagnostics GmbH (Mannheim, Germany). Hepatic triglycerides were extracted by a heptane-isopropanol-Tween mixture (3:2:0.01 by volume) and determined with the commercial kits mentioned above.

### Statistical Analysis

All results were expressed as the mean ± SEM. Statistical analysis was performed with a two-tailed unpaired *t*-test. *p* < 0.05 was considered statistically significant.

## Results

### Linderane Inhibited Gluconeogenesis in Rat Primary Hepatocytes

**FIGURE 1:**
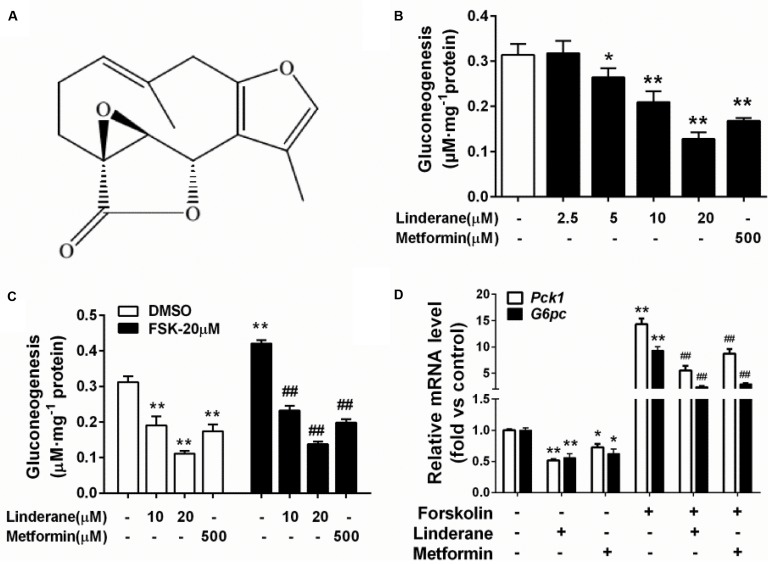
Effect of linderane on gluconeogenesis in rat primary hepatocytes. **(A)** Chemical structure of linderane. **(B)** Dose-dependent suppression of gluconeogenesis by linderane in rat primary hepatocytes. Cultured hepatocytes were incubated with 10 or 20 μM linderane, and gluconeogenesis was detected. **(C)** Expression of *Pck1* (white bars) and *G6pc* (black bars). **(D)** With or without forskolin stimulation. In these experiments, metformin was used as a positive control. All results are presented as the mean ± SEM (*n* = 4). ^∗^*p* < 0.05, ^∗∗^*p* < 0.01 versus control under basal conditions; ^##^*p* < 0.01 versus control under forskolin-stimulated conditions.

The structure of linderane, a natural product, is shown in **Figure 1A**. The gluconeogenesis in rat primary hepatocytes was suppressed by linderane in a dose-dependent manner (**Figure 1B**), with 10 and 20 μM linderane resulting in a decrease of 39.8 and 65.6%, respectively. Linderane at 20 μM showed a comparable effect with 500 μM metformin under basal conditions. Forskolin, an AC activator, significantly stimulated gluconeogenesis in primary hepatocytes. Linderane exerted an inhibitory effect on forskolin-stimulated gluconeogenesis, with 10 and 20 μM linderane causing a reduction by 44.1 and 71.7%, respectively, which were similar to the effect of linderane under basal conditions (**Figure 1C**). Moreover, 20 μM linderane significantly decreased the mRNA expression levels of *Pck1* and *G6pc*, two key gluconeogenic genes, under both basal and forskolin-stimulated state (**Figure 1D**).

### Linderane Suppressed cAMP/PKA/CREB Pathway in Rat Primary Hepatocytes

**FIGURE 2:**
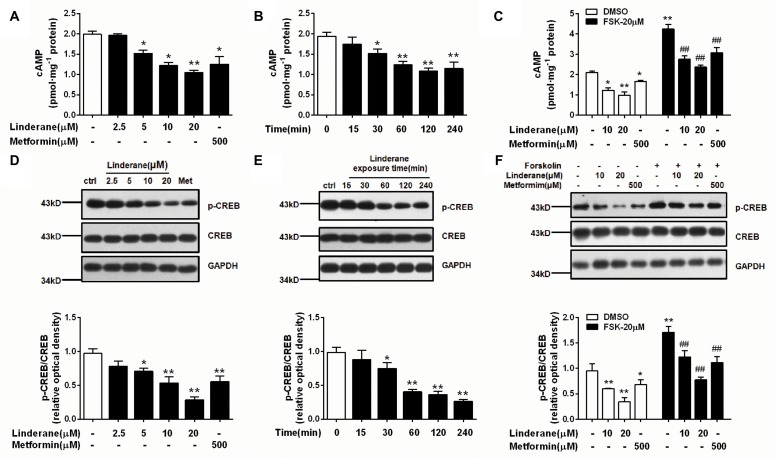
Effect of linderane on the cAMP pathway in rat primary hepatocytes. **(A,D)** Reduced cAMP content and decreased CREB phosphorylation by different doses of linderane after incubation for 2 h. **(B,E)** Reduced cAMP content and decreased CREB phosphorylation by 20 μM linderane after incubation for different times. **(C,F)** Reduced cAMP content and decreased CREB phosphorylation by 10 or 20 μM linderane with or without forskolin stimulation. In these experiments, metformin was used as a positive control. All results are presented as the mean ± SEM (*n* = 3–4). ^∗^*p* < 0.05, ^∗∗^*p* < 0.01 versus control under basal conditions; ^##^*p* < 0.01 versus control under forskolin-induced conditions.

Intracellular cAMP content and CREB phosphorylation were measured in rat primary hepatocytes after incubation with linderane. Linderane dose- and time-dependently decreased the cAMP concentration in hepatocytes. Treatment with 10 and 20 μM linderane for 2 h reduced cAMP concentration by 38.5 and 47.2%, respectively. Furthermore, 20 μM linderane presented a comparable potency with 500 μM metformin (**Figure 2A**). The inhibitory effect of 20 μM linderane on cAMP content appeared within 30 min and continued up to 240 min (**Figure 2B**). Reduction in forskolin-induced cAMP accumulation was also observed after linderane treatment. Linderane at 10 and 20 μM decreased cAMP levels by 35.0 and 44.1%, respectively (**Figure 2C**). This effect was similar to that under basal conditions. Correspondingly, the phosphorylation of CREB was suppressed by linderane under both basal and forskolin-stimulated conditions (**Figures 2D–F**).

### Linderane Indirectly Activated Phosphodiesterase 3

**FIGURE 3:**
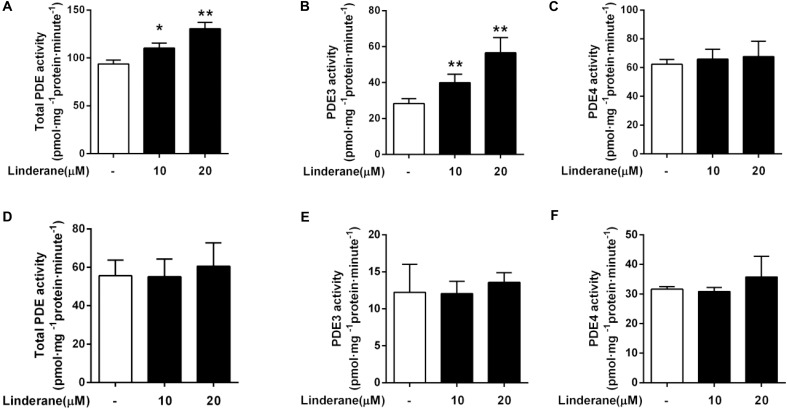
Effect of linderane on PDE activity. Cell lysates were prepared from the cultured hepatocytes to detect the indirect activities of total PDEs **(A)**, PDE3 **(B)**, and PDE4 **(C)**. Purified PDE enzymes were applied to detect the direct activities of total PDEs **(D)**, PDE3 **(E)**, and PDE4 **(F)**. All results are presented as the mean ± SEM (*n* = 3). ^∗^*p* < 0.05, ^∗∗^*p* < 0.01 versus control.

We detected the effect of linderane on PDE activity in primary rat hepatocytes. As shown in **Figure 3A**, 10 and 20 μM linderane treatment increased total PDE activity by 17.6 or 39.2%, respectively. Linderane at 10 and 20 μM increased PDE3 activity in cultured hepatocytes by 41.1 and 99.5%, respectively (**Figure 3B**), whereas PDE4 activity was not altered (**Figure 3C**). The data indicated that linderane activated PDE3 in hepatocytes. We further assessed the direct effect of linderane on PDE activity using purified PDE enzymes. Linderane did not alter the activity of total PDEs (**Figure 3D**), PDE3 (**Figure 3E**), and PDE4 (**Figure 3F**), suggesting that linderane activated PDE3 in an indirect way.

### Linderane Inhibited Gluconeogenesis by Activating Phosphodiesterase 3 in Rat Primary Hepatocytes

**FIGURE 4:**
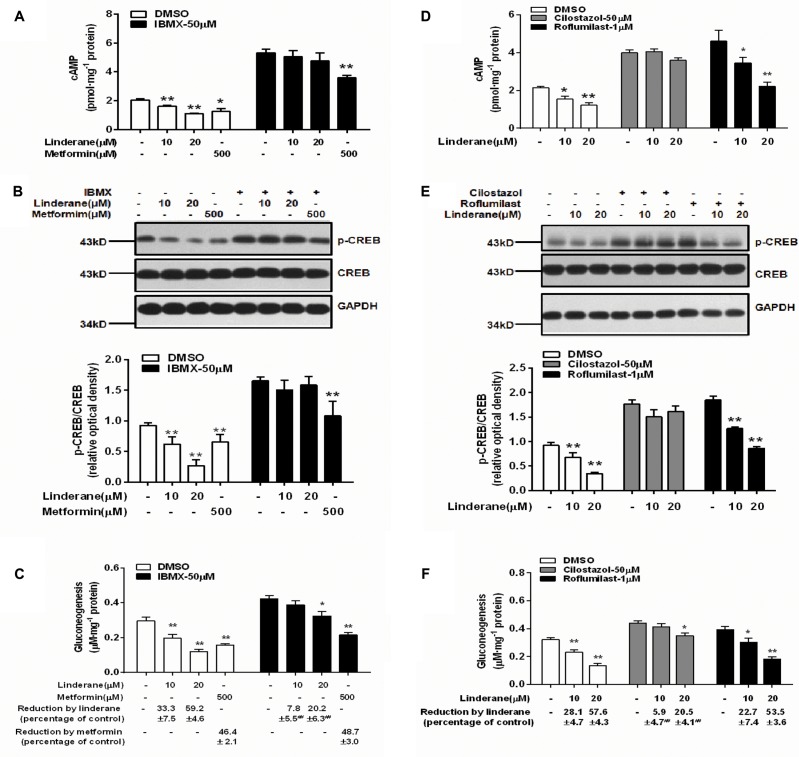
Linderane inhibited gluconeogenesis by activating PDE3 in rat primary hepatocytes. Cultured hepatocytes were pretreated with or without 50 μM IBMX for 30 min and co-treated with 10 μM linderane, 20 μM linderane, or 500 μM metformin. cAMP concentration **(A)**, CREB phosphorylation **(B)**, and gluconeogenesis **(C)** were detected. Cultured hepatocytes were pretreated with a PDE3 inhibitor (cilostazol, 50 μM) or PDE4 inhibitor (roflumilast, 1 μM) for 30 min and co-treated with 10 or 20 μM linderane. cAMP concentration **(D)**, CREB phosphorylation **(E)**, and gluconeogenesis **(F)** were detected. All results are presented as the mean ± SEM (*n* = 3–4). ^∗^*p* < 0.05, ^∗∗^*p* < 0.01 versus individual control; ^##^*p* < 0.01 versus reduction caused by linderane under basal conditions (shown as percentage of control).

To identify whether linderane inhibited gluconeogenesis through PDE activation and cAMP suppression, IBMX was used as a pan-PDE inhibitor. In the presence of 50 μM IBMX, the reduction in cAMP accumulation and CREB phosphorylation induced by 10 or 20 μM linderane was abolished (**Figures 4A,B**). Moreover, the gluconeogenesis inhibition caused by 10 μM linderane was fully blocked, and the reduction in gluconeogenesis caused by 20 μM linderane was also significantly diminished (59.2% vs. 20.2%, without or with IBMX, *p* < 0.01, **Figure 4C**). A specific PDE3 inhibitor (cilostazol) and a specific PDE4 inhibitor (roflumilast) were applied in the following experiments. As shown in **Figures 4D,E**, the reduction in cAMP accumulation and CREB phosphorylation induced by 10 or 20 μM linderane was completely blocked by cilostazol but not roflumilast. Moreover, the inhibitory effect of linderane on gluconeogenesis was also significantly attenuated in the presence of cilostazol but not roflumilast (**Figure 4F**). These data suggested that linderane suppressed hepatic gluconeogenesis mainly through PDE3 activation.

### Linderane Indirectly Activated PDE3 Through ERK/STAT3 in Rat Primary Hepatocytes

**FIGURE 5:**
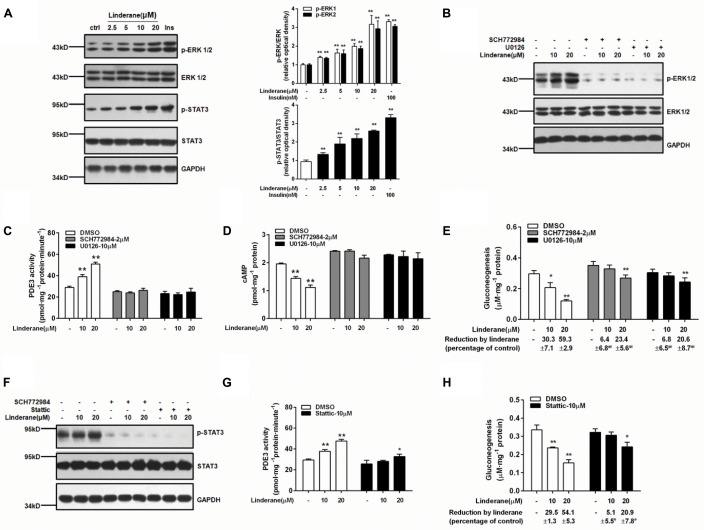
Linderane indirectly activated PDE3 through ERK/STAT3 in rat primary hepatocytes. **(A)** Dose-dependent activation of ERK1/2 and STAT3 induced by linderane after 2 h of incubation. Insulin (Ins, 100 nM) was used as a positive control. Cultured hepatocytes were preincubated with or without an ERK1/2 inhibitor (SCH772984, 2 μM) or an MEK inhibitor (U0126, 10 μM) for 30 min and then were co-treated with 10 or 20 μM linderane. ERK1/2 phosphorylation **(B)**, PDE3 activity **(C)**, cAMP content **(D)**, and gluconeogenesis **(E)** were detected. The ERK1/2 inhibitor (SCH772984, 2 μM) and STAT3 inhibitor (Stattic, 10 μM) abolished the increased phosphorylation of STAT3 by linderane **(F)**. Cultured hepatocytes were preincubated with or without Stattic for 30 min and then were co-treated with 10 or 20 μM linderane. PDE3 activity **(G)** and gluconeogenesis **(H)** were detected. All results are presented as the mean ± SEM (*n* = 3–4). ^∗^*p* < 0.05, ^∗∗^*p* < 0.01 versus individual control; ^#^*p* < 0.05, ^##^*p* < 0.01 versus reduction caused by linderane under basal conditions (shown as percentage of control).

Linderane increased the ERK1/2 and STAT3 phosphorylation in rat primary hepatocytes (**Figure 5A**). Both the ERK1/2 inhibitor SCH772984 (2 μM) and MEK inhibitor U0126 (10 μM) abrogated the effect of linderane on PDE3 activity and cAMP accumulation (**Figures 5B–D**). Meanwhile, the reduction in gluconeogenesis induced by linderane was significantly restored (**Figure 5E**). The ERK1/2 inhibitor and a STAT3 inhibitor Stattic (10 μM) attenuated the STAT3 phosphorylation by linderane (**Figure 5F**). Furthermore, in the presence of Stattic, the majority effect of linderane on PDE3 activity (**Figure 5G**) and gluconeogenesis (**Figure 5H**) was abolished.

### Linderane Activated Hepatic PDEs and Suppressed the cAMP/PKA/CREB Pathway in C57BL/6J Mice

**FIGURE 6:**
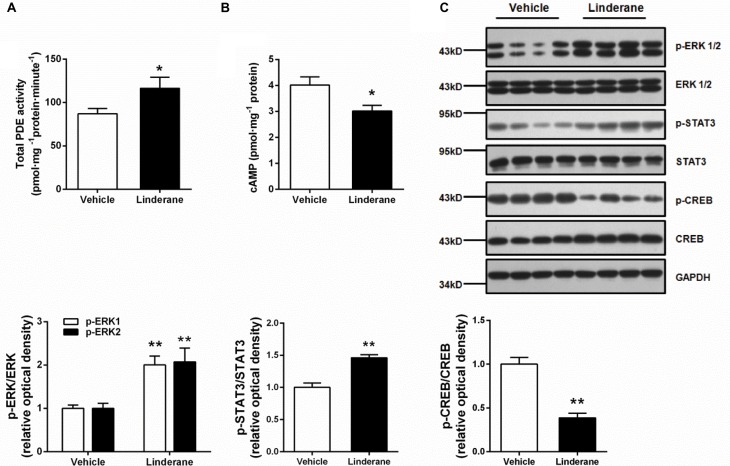
Effect of linderane on total PDE activity and the cAMP pathway in C57BL/6J mice. Linderane (50 mg kg^-1^) or vehicle was orally administered in C57BL/6J mice. Total PDE activity **(A)** and cAMP content **(B)** were determined in the liver using commercial kits as described above. **(C)** ERK1/2, STAT3, and CREB phosphorylation were detected in the liver using western blot analysis. All results are presented as the mean ± SEM (*n* = 8). ^∗^*p* < 0.05, ^∗∗^*p* < 0.01 versus vehicle mice.

The *in vivo* effect of linderane (50 mg kg^-1^) on hepatic PDE activity and the cAMP pathway was evaluated in C57BL/6J mice. The total PDE activity in the liver was elevated by 33.7% after administration of linderane (**Figure 6A**). Correspondingly, linderane treatment reduced the cAMP concentration (**Figure 6B**) and CREB phosphorylation level in the liver (**Figure 6C**). In addition, linderane treatment increased the phosphorylation of ERK1/2 and STAT3 (**Figure 6C**). These data showed that linderane could activate hepatic PDEs through ERK/STAT3 and thus suppress cAMP signaling pathway *in vivo*.

### Chronic Linderane Treatment Ameliorated Metabolic Abnormalities in *ob/ob* Mice

**FIGURE 7:**
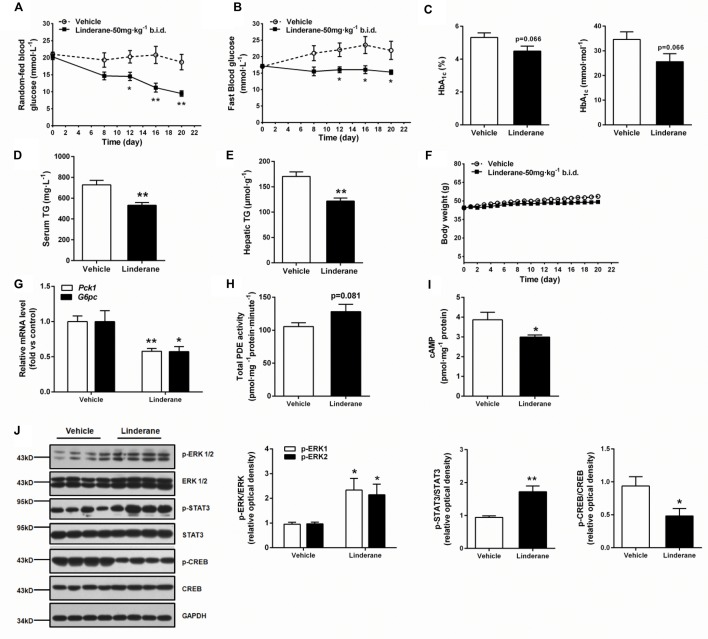
Effect of chronic linderane treatment on metabolic disorders in *ob/ob* mice. Male *ob/ob* mice were treated with linderane (50 mg kg^-1^, twice a day, p.o.) or vehicle for 20 days. Random-fed **(A)** and fast blood-glucose levels **(B)** were detected on day 8, 12, 16, and 20. Serum HbA_1c_ **(C)** and serum triglyceride levels **(D)** were determined after the treatment. **(E)** Mouse livers were collected to detect triglyceride content. **(F)** Body weight was regularly recorded. **(G)** Expression of *Pck1* (white bars) and *G6pc* (black bars) was determined in the liver. Total PDE activity **(H)** and cAMP content **(I)** were evaluated using commercial kits. **(J)** ERK1/2, STAT3, and CREB phosphorylation was detected in the liver using western blot analysis. All results are presented as the mean ± SEM (*n* = 7–8). ^∗^*p* < 0.05, ^∗∗^*p* < 0.01 versus vehicle mice.

The chronic glucose-lowering potential of linderane (50 mg kg^-1^, twice a day) was evaluated in male *ob/ob* mice. Treatment with linderane significantly decreased random-fed and fast blood glucose levels with a reduction rate of 49.4 and 30.2%, respectively, after 20 days of treatment (**Figures 7A,B**). The long-term glycemic indicator HbA_1c_ tended to be reduced by linderane treatment (*p* = 0.066, shown as percentage or mmol mol^-1^) (**Figure 7C**). Furthermore, serum triglyceride levels and hepatic triglyceride contents were significantly decreased by 26.9 and 28.5%, respectively (**Figures 7D,E**). The body weight did not change during the treatment (**Figure 7F**). Linderane also caused a 42.2 and 42.6% decrease in the mRNA expression levels of hepatic *Pck1* and *G6pc*, respectively (**Figure 7G**). Long-term treatment with linderane increased the total PDE activity in the liver by 28.3% (*p* = 0.081) (**Figure 7H**) and decreased cAMP concentration by 22.6% (**Figure 7I**). In addition, linderane increased ERK1/2 and STAT3 phosphorylation and decreased CREB phosphorylation (**Figure 7J**) in the liver.

## Discussion

The liver is the predominant site for whole-body glucose homeostasis. As the duration of fasting increases, the proportion of gluconeogenesis in hepatic glucose production increases rapidly (Bano, 2013). Furthermore, abnormally enhanced hepatic gluconeogenesis in T2DM contributes to chronic hyperglycemia (Gray et al., 2015). In the discovery of new strategies for glycemic control, targeting the cAMP pathway for gluconeogenesis inhibition has attracted much attention in recent years (Petersen et al., 2017). In this study we revealed that the natural product linderane could suppress gluconeogenesis by inhibiting the cAMP pathway through activation of a cAMP-degrading enzyme, PDE3 in rat primary hepatocytes. The upstream signaling regulation of PDE3 activation by linderane was also investigated in the present study.

In hepatocytes, cellular cAMP accumulation triggers PKA activation and induces CREB phosphorylation, which further leads to the formation of the CREB-CBP-CRTC2 complex, resulting in the elevation of gluconeogenesis (He et al., 2009). In this study, linderane was discovered to inhibit gluconeogenesis and reduce the intracellular levels of cAMP in rat primary hepatocytes. Since the phosphorylation of CREB Ser 133 is considered an important factor in cAMP pathway activation (Pernicova and Korbonits, 2014), we assessed the effect of linderane on CREB phosphorylation. As expected, CREB phosphorylation was significantly inhibited by linderane treatment. Thus, the inhibition of gluconeogenesis by linderane in rat hepatocytes might be mediated by the suppression of cAMP signaling pathway.

The second messenger cAMP controls multiple essential cellular functions. Physiologically, the intracellular cAMP level is strictly maintained within a narrow range. cAMP is generated by AC from ATP and terminated by the PDE enzymes, which function by catalyzing hydrolysis of the cyclic phosphate bond of cAMP and then inactivate the cAMP pathway (Furman et al., 2010; Francis et al., 2011). We found that linderane increased total PDE activity in cultured hepatocytes. In the PDE superfamily, the PDE3 and PDE4 families were chiefly responsible for cAMP degradation in rat primary hepatocytes (Hermsdorf and Dettmer, 1998), even though the expression of several other PDE families was also detected in the liver (Kanageswaran et al., 2015). Linderane significantly increased the activity of PDE3 in rat hepatocytes but not that of purified PDE enzymes. Thus, linderane-induced inhibition of the cAMP pathway might occur primarily through indirect PDE3 activation.

The role of PDE3 in hepatic glucose metabolism is poorly elucidated, although a few studies have suggested its ability to maintain hepatic energy through cAMP pathway antagonism. Whole-body depletion of PDE3B (one important subfamily of PDE3) in mice caused excessive cAMP accumulation in liver, which resulted in abnormal gluconeogenic gene expression and systemic glucose dysregulation (Choi et al., 2006). However, as PDE3B is also expressed in metabolic tissues such as adipose, the contribution of hepatic PDE3B to overall glucose homeostasis could not be directly observed in the PDE3B knockout model (Sahu et al., 2017). Moreover, no selective small molecule activators of PDE3 have yet become available to assess the potential of PDE3 for regulating energy balance or explore its upstream signaling. Here we proved that linderane could act as a molecular tool to activate PDE3 in hepatocytes. The pan-PDE inhibitor IBMX and PDE3 specific inhibitor cilostazol attenuated the gluconeogenesis-suppressive effect of linderane in the rat primary hepatocytes, suggesting that linderane activated PDE3 and subsequently inhibited gluconeogenesis.

Given that linderane did not increase PDE3 activity directly, the upstream regulators were investigated. To our knowledge, few studies so far elucidated the upstream molecular regulation of PDE3 in the liver, especially PDE3-dependent gluconeogenesis inhibition. An inorganic compound, orthovanadate, was reported to stimulate PDE3 through p44 MAPK in rat primary hepatocytes (Watanabe et al., 2004). Furthermore, activation of constitutive MEK-ERK in mouse primary hepatocytes resulted in an increase in PDE activity (Ramakrishnan et al., 2016). We discovered that linderane activated ERK1/2 in cultured hepatocytes. Moreover, both pharmacological MEK and ERK1/2 inhibitors restored the PDE3 activation and cAMP suppression induced by linderane, demonstrating that ERK1/2 might participate in the regulation of PDE3. As ERK1/2 controls a range of important cellular functions (Unal et al., 2017), the precise mechanism by which ERK1/2 activates PDE3 was further explored. STAT3 was shown to be activated by ERK1/2 in EGF-dependent signaling, which defined STAT3 as a downstream of ERK1/2 (Lo, 2010). In our study, linderane activated STAT3 in primary hepatocytes and this effect was abrogated by an ERK1/2 inhibitor SCH772984, showing that ERK1/2 acted as an upstream regulator of STAT3 under the linderane-treatment conditions. One recent study reported that in mouse hypothalamus, STAT3 activation was observed in PDE3B-expressing neurons after leptin treatment (Sahu and Sahu, 2015). We theorized that STAT3 played a role in PDE3 activation by linderane. As expected, a STAT3 inhibitor Stattic abolished the STAT3 activation and restored a majority of the PDE3 activation induced by linderane, suggesting that STAT3 was required for linderane to activate PDE3 through ERK1/2 in hepatocytes. These findings were further supported by the *in vivo* study. A single oral administration of linderane in C57 mice increased the phosphorylation of ERK1/2 and STAT3 in the liver, which led to the elevation of PDE activity and suppression of cAMP/CREB pathway. Taken together, our results indicated that linderane activated PDE3 through the ERK/STAT3 pathway, which provided more detailed evidence for research on hepatic PDE3 regulation.

In diabetic patients, aberrant hepatic gluconeogenesis during fasting disrupts the whole-body glucose balance and contributes to persistent hyperglycemia. This effect is at least partly triggered by abnormal enhancement in the cAMP/PKA/CREB pathway (Taylor, 1999). Chronic administration of linderane increased hepatic PDE activity, as well as the phosphorylation of ERK1/2 and STAT3. These findings implied that linderane could activate PDEs through ERK/STAT3, which ultimately lead to hepatic gluconeogenesis inhibition. Linderane significantly reduced both random-fed and fast glucose levels, indicating its ability to improve whole-body glucose metabolism. This amelioration was further confirmed by the fact that chronic linderane treatment caused a decreased serum HbA_1c_ level, which acts as an important glycemic indicator in blood glucose control. Apart from glucose metabolism, linderane could reduce serum and hepatic triglyceride levels, which might occur secondarily to hyperglycemia correction during long-term treatment. Overall, we demonstrated that hepatic PDE activation by linderane could significantly suppress gluconeogenesis and improve blood glucose and lipid metabolism in the diabetic *ob/ob* mouse model.

## Conclusion

We presented the beneficial effect of linderane on diabetic disorders and its underlying molecular mechanism. Linderane indirectly activated PDE3 through ERK/STAT3 activation and subsequently suppressed the cAMP/PKA/CREB pathway, leading to gluconeogenesis inhibition in primary hepatocytes. Chronic administration of linderane decreased blood glucose levels in *ob/ob* mice. These findings highlighted the prospect of hepatic PDE3 for maintaining whole-body energy homeostasis and provided further information for the development of hepatic PDE3 as a potential target for T2DM therapeutics.

## Author Contributions

YL, JS, and SH designed the research. WX, YY, YF, TX, and SH performed the research. WX, SH, JS, and YL analyzed and interpreted the data. WX, SH, and YL wrote the paper. All authors gave their final approval of the version to be published.

## Conflict of Interest Statement

The authors declare that the research was conducted in the absence of any commercial or financial relationships that could be construed as a potential conflict of interest.

## Abbreviations

AC: adenylate cyclase
AMPK: AMP-activated protein kinase
cAMP: cyclic adenosine monophosphate
CREB: cAMP-response element-binding protein
CRTC2: CREB-modulated transcription co-activator 2
ERK1/2: Extracellular regulated protein kinase 1/2
G6pc: glucose-6-phosphatase
HbA1c: hemoglobin A1c
IBMX: 3-isobutyl-1-methylxanthine
Pck1: phosphoenolpyruvate carboxykinase
PDE: phosphodiesterase
PKA: protein kinase A
STAT3: signal transducer and activator of transcription 3
T2DM: type 2 diabetes mellitus
